# siRNA Delivery Improvement by Co-formulation of Different Modified Polymers in Erythroleukemic Cell Line K562

**Published:** 2013-09

**Authors:** Mazdak Ganjalikhani hakemi, Maryam Hashemi

**Affiliations:** 1 Cellular & Molecular Immunology Research Center, Isfahan University of Medical Sciences, Isfahan, Iran; 2 Immunology Department, Faculty of Medicine, Isfahan University of Medical Sciences, Isfahan, Iran; 3 Nanotechnology Research Center, School of Pharmacy, Mashhad University of Medical Sciences, Mashhad, Iran

**Keywords:** OEI, PEI, siRNA delivery, Suspended cells, Transferrin

## Abstract

***Objective(s):*** siRNA may be a very promising tool for treatment of various diseases especially in cancer therapy due to high specificity. One of the main hurdles applications of siRNAs *in vivo* is optimization of the delivery strategy, especially the carrier systems. The aim of this study was to optimize siRNA delivery into suspended erythroleukemic cell line K562.

***Materials and Methods:*** We applied polyethyleneimine (PEI) and oligoethyleneimine (OEI) derivatives alone or their co-formulation with different agents such as chloroquine (a drug known to alter lysosomal pH and thus to inhibit lysosomal degradation of macromolecules), DOPE (lipophilic agent), succinic acid (introduction of negatively charged to polymer) and transferrin (the ligand of transferring receptor which is over-expressed in many types of tumors and hematopoietic cells).

***Results:*** In this study it was shown that utilizing a combination of 70% OEI-HA10 (ten hexyl acrylate residues per one OEI chain) plus 30% of transferin-PEI with Luc-siRNA was highly effective for transfecting K562 cell. This co-formulation silenced luciferase activity up to 70% after short time without any significant inhibition in the luciferase activity in siCONTROL wells.

***Conclusion:*** In conclusion, the combination of modified PEI with transferrin and OEI by hexyl acrylate may increase siRNA delivery and reduce toxicity in hematopoietic suspended cells.

## Introduction

The discovery of RNA interference (RNAi) by Andrew Fire and colleagues in 1998 provided an unexpected new approach for basic and applied biology (1). RNAi has extremely high and specific inhibitory effect on the gene expression in mammalian cells (2-6). Although therapeutic RNAs have the potential to revolutionize medicine, until now naked RNA formulations have only been administered successfully to local tissues by direct injection, e.g. into the eye for the treatment of adult late stage wet macular degeneration (AMD) (7). The clinical use of this technology requires easy-to-handle systemic applications to address a broad range of disease indications. For this purpose, safe additional carrier systems need to be developed, to protect the RNA in the extracellular environment and effectively deliver it to the site of interest. Recently, several strategies for the development of polymeric carriers were introduced, and are mainly based on optimization of the conventional vectors for DNA delivery (7-9). These structures formed polyplexes with siRNA, to increase stability against dissociation. Structures with enhanced endosomolytic properties were more efficient in siRNA delivery. 

Polyethylenimine (PEI) is a polycationic carrier for nucleic acids with intrinsic/inherent endosomal activity, mediating high DNA transfection activity *in vitro *and also *in vivo*. *In vivo* application, however, is limited by a significant toxicity and lack of biodegradability of the polymer (10, 11). To develop polycationic carriers are as effective as the golden standard PEI, but less toxic and biodegradable new polymers. The development was performed based on low molecular weight oligoethylenimines (OEI). 

Hydrophobically modified OEI 800 by Michael, in the addition of alkyl acrylates was assessed for siRNA delivery in murine neuroblastoma cells (Neuro2A/EGFPLuc), human hepatoma cells (HUH7/EGFPLuc), which are stably transfected with the EGFPLuc gene, or human lung carcinoma cells H1299/Luc stably transfected with the luciferase gene (8). Among different OEI formulations, the structure containing 10 hexyl acrylate residues per one OEI chain (OEI-HA-10) was the only effective oligoamine for siRNA delivery which induced efficient knockdown (8). 

Co-formulation of polymers with different agents could improve the efficiency of the carrier and decrease its toxicity (12-14). Specific tissue targeting is essential for effective *in vivo* nucleic acid delivery and low side effects. The transferrin (Tf) receptor, a cell surface receptor for the uptake of the glycoprotein Tf, is over-expressed in many types of tumors and hematopoietic cells. Several studies have utilized the polymer and Tf receptor for targeting their DNA or RNA vehicles (12, 15-18). 

The aim of this study was to optimize siRNA delivery into erythroleukemic tumor cells K562 which over express the transferrin receptor. PEI and oligoethyleneimine were used alone or in combination with their different derivatives (Previously synthesized in Professor Wagner’s lab, Pharmaceutical Biotechnology, Ludwig Maximilians University, Munich, Germany). In this study, it was hypothesized that this method may increase siRNA delivery and reduces toxicity of polymers.

## Materials and Methods

Linear PEI (22 kDa) and Tf-PEI (transferring conjugated to 25 kDa branched PEI) were prepared as previously described (17), suc-PEI as described (18), OEI-HA10 and OEI-HA10/DOPE as described (8). The plasmid pEGFPLuc (Clontech Laborato-ries, Heidelberg, Germany) containing a CMV promoter driven fusion of the genes encoding for enhanced green fluorescent protein and luciferase was used for generation of stably transfected K562 cells. Oligoethylenimine (OEI) with an average molecular weight of 800 Da and all other chemicals were purchased by Sigma-Aldrich (Munich, Germany). Cell culture media, antibiotics, and fetal calf serum (FCS) were purchased from Invitrogen (Karlsruhe, Germany). RNase-free water, absolute ethanol and dimethyl sulfoxide puriss (DMSO) were obtained from Sigma-Aldrich (Munich, Germany). Luciferase cell culture lysis buffer and D-luciferin sodium salt were obtained from Promega (Mannheim, Germany). Ready to use siRNA duplexes were purchased from Dharmacon (Lafayette, CO), namely, luciferase-siRNA: GL3 luciferase duplex: 5′-CUUACGCUGAGUAC-UUCGAdTdT-3′ (sense); control-siRNA (siCONTROL): nontargeting control duplex: 5′-AUGUAUUGGCCUGUAUUAGUU-3′ (sense). siCONTROL is a validated, nontargeting siRNA specially designed to have no gene targets in human, mouse, and rat cells. It also shows minimal off-target effects and at least four mismatches with all known human, mouse, and rat genes and is therefore recommended by Dharmacon as a negative control siRNA to distinguish sequence-specific from nonspecific targeting.

***Polyplex formation***

In all studies, the composition of polyplexes was characterized by the w/w ratio of the polymer to nucleic acid in the mixture. The weight of Tf-PEI, suc-PEI and OEI-HA10 represents the weight of lyophilized product, which was previously neutralized and, therefore, includes the mass of counter ions (chloride). Polyplex formulations for siRNA delivery were prepared in HBG (HEPES buffered glucose solution; 20 mM HEPES, 5% glucose, pH 7) as follows: Different concentrations of formulations were diluted at various polymer/nucleic acid ratios in separate tubes in HBG (20 μl). Then, the HBG solution of oligoamine was added to the HBG solution of the nucleic acid, mixed by pipetting up and down and incubated for 20-30 min at room temperature to form the siRNA polyplexes that were used for transfection experiments.

***Cell culture***

All cultured cells were grown at 37°C in 5% CO_2 _humidified atmosphere. K562 cells/EGFPLuc cells were grown in RPMI1640 medium (4.5 g/l glucose) supplemented with 10% FCS, 4 mM stable glutamine, 100 U/ml penicillin, and 100 μg/ml streptomycin. 

***Luciferase gene silencing***

 Cells were seeded in 48-well plates (TPP, Trasadingen, Switzerland) 24 hr prior to transfection using 500000 cells per well. Then transfection complexes containing siRNA (5 μg) were added to the cells in 500 μl of culture medium containing 10% serum, after different times (24 hr, 4 hr, 1 hr and 40 min) medium was removed and cells were lysed in 100 μl of Promega cell lysis solution for 20 min at room temperature to measure the gene expression. Luciferase activity was measured using a Lumat LB9507 instrument (Berthold, Bad Wildbad, Germany). Transfection efficiency was evaluated as relative light units (RLU) per number of seeded cells. Transfections were also performed with a nonspecific control siRNA to distinguish between specific gene silencing and unspecific knockdown of protein expression due to carrier toxicity. 

For Tf competition experiments, free Tf (1.25 µl/mg ferrous citrate in 50 mg/ml transferrin solution) in a final concentration of 1 μg/μl was added to the cells 15 min prior to transfection and medium change was performed 40 min following siRNA delivery. Untreated transfected cells were set as control for competition experiments.

***Statistical analysis***

Results are presented as mean and relative SD, and statistical significance of differences was evaluated by Paired Sample T test: p values smaller than 0.05 were considered to be statistically significant, * *P* < 0.05.

## Results

In the current study, we evaluated the effectiveness of different new synthesized polymers for transfecting K562 cell line, as a representative of hematopoietic suspended cells, with siRNA. These experiments were performed either with single polymer or with a co-formulation of two distinct polymers in different ratios. K562 cells were stably transfected with DNA encoding luciferase (K562 eGFP-Luc) and hence, siRNA specific for luciferase mRNA (Luc-siRNA) was utilized to inhibit chemiluminescent activity of the cells in order to reveal transfection efficiencies. Various w/w ratios of polymers to siRNA have been used.

**Figure 1:**
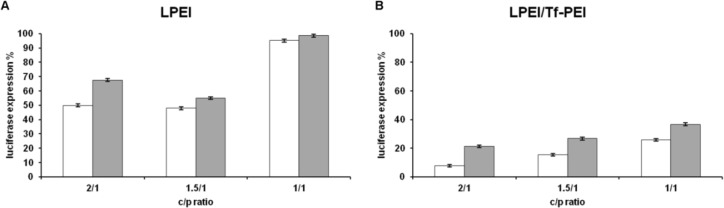
Knocking down of luciferase activity in K562 cells after 24 hr incubation with Luc-siRNA formulations. (A) LPEI and (B) LPEI (80%) +Tf-PEI (20%) tested at 1/1, 1.5/1 and 2/1 w/w ratios with Luc-siRNA as indicated under the x-axis. White bars represent luciferase activity (%) in comparison to untreated control cells (100%) obtained by using Luc-siRNA and gray bars represent activities obtained with siCONTROL. Data are mean and relative SD of 5 similar experiments

As it is shown in Figure 1, applying linear PEI neither alone nor in combination with Tf-PEI was successful in transfecting K562 cells with luciferase specific siRNA. The maximum silencing efficiency of linear PEI was 52% while 45% silencing was observed in siCONTROL containing wells which represent toxicity rather than specific silencing (Figure 1A). The maximum effect following siRNA transfection with linear PEI plus Tf-PEI was 71% and 72% for Luc-siRNA and siCONTROL wells, respectively (Figure 1B). This effect was rather toxic as well.

**Figure 2:**
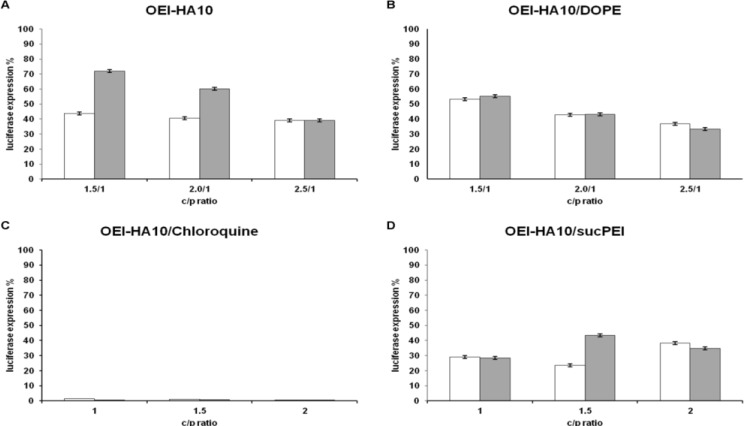
Suppression of luciferase activity in K562 cells after 24 hr incubation with Luc-siRNA formulations. A) OEI-HA10, B) OEI-HA10/DOPE (1.5-2.5/1 w/w ratio), C) OEI-HA10 (1-2/1 w/w ratio) and chloroquine (1/1 w/w ratio), and D) OEI-HA10 (1-2/1 w/w ratio) and sucPEI (1/1 w/w ratio). W/w ratios with Luc-siRNA are indicated under the x-axis. White bars represent luciferase activity (%) in comparison to untreated control cells (100%) obtained by using Luc-siRNA and gray bars represent activities obtained with siCONTROL. Data are mean and relative SD of 5 similar experiments

Maximum inhibitory effect of Luc-siRNA formulations with OEI-HA10 on luciferase activity of K562 cell line was 61%. However, 61% decrease in this luciferase activity in siCONTROL wells represents a total toxic effect on cells (Figure 2A). The results of applying combinations of OEI-HA10 with helper lipid DOPE with different w/w ratios were similar to using OEI-HA10 alone, with 63% silencing with Luc-siRNA and 67% silencing with siCONTROL (Figure 2B). Combinations of OEI-HA10 with either malaria drug chloroquine, a drug known to alter lysosomal pH and thus to inhibit lysosomal degradation of macromolecules, (Figure 2C) or suc- PEI (Figure 2D) resulted in much more cytotoxic effects on K562 cells. 

**Figure 3:**
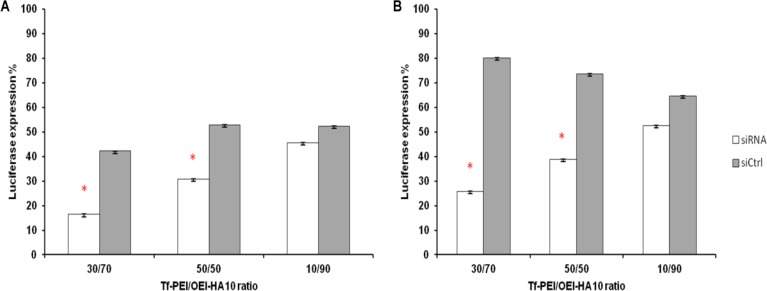
Suppression of luciferase activity in K562 cells after incubation with Luc-siRNA formulations with Tf-PEI and OEI-HA10. A) Luciferase activity after 24 hr of incubation and B) after 1 hr of incubation with Tf-PEI/OEI-HA10 co-formulations. Different ratios of Tf-PEI/OEI-HA10 co-formulation are indicated in X axis. White bars represent luciferase activity (%) in comparison to untreated control cells (100%) obtained by using Luc-siRNA and gray bars represent activities obtained with siCONTROL. Data are mean and relative SD of 5 similar experiments

In the next step, different ratios of OEI-HA10 and Tf-PEI with Luc-siRNA were examined. After 24 hr, 84% silencing was observed using 70% and 30% OEI-HA10 and Tf-PEI respectively, but the luciferase activity was 58% suppressed in siCONTROL sample as well (Fgure 3A). Reducing the incubation time, After 1 hr incubation 74% of luciferase activity was suppressed, but there was still 20% silencing in siCONTROL transfected cells (Figure 3B). The amount of knockdown was also measured after 4 hr which had the same results as 24 hr incubation (data not shown). 

**Figure 4:**
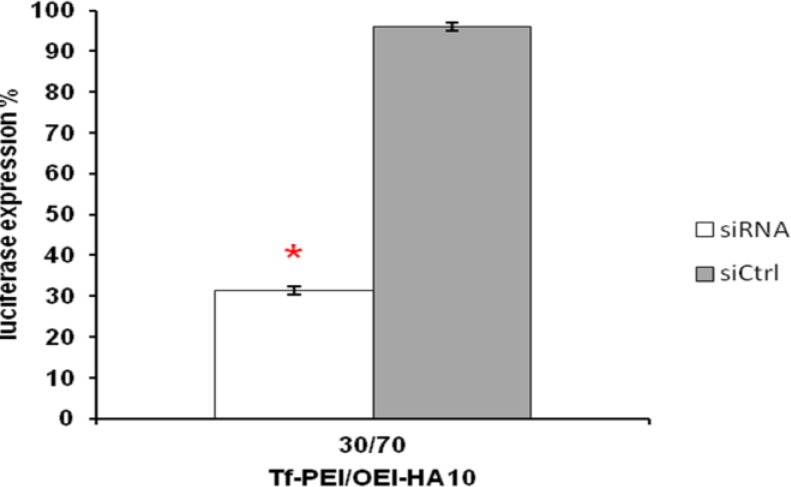
Suppression of luciferase activity in K562 cells after incubation with OEI-HA10/Tf-PEI/Luc-siRNA formulation. 40 min incubation of K562 cells with OEI-HA10 (70%)/Tf-PEI (30%)/Luc-siRNA formulation in 0.5/1 w/w ratio, showed a significant suppression in luciferase activity. White bar represents luciferase activity (%) in comparison to untreated control cells (100%) obtained by using Luc-siRNA and gray bar represents activities obtained with siCONTROL. Data are mean and relative SD of 5 similar experiments. Asterisk indicates statistical significance compared to control treated cells (*P<0.05; paired-sample t- test

Best result was obtained with 70% OEI-HA10 plus 30% of Tf-PEI at c/p ratio 0.5 after 40 min incubation at 37°C and 5% CO_2_. The silencing effect on luciferase activity in this case was 69% without any significant knocking down in siCONTROL transfected cells (Figure 4).

**Figure 5:**
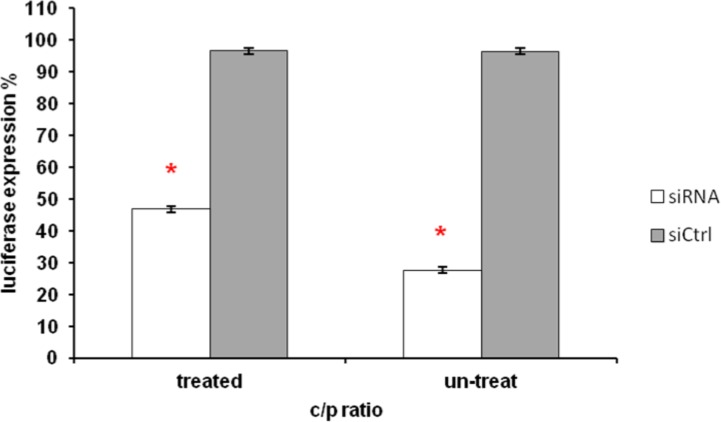
Competition assay. One group of cultured K562 cells was treated with ferrous citrate in transferrin solution 15 min prior to transfection with OEI-HA10/Tf-PEI/Luc-siRNA formulation and the other was not. Cells un-treated with transferrin solution, showed a significant suppression in luciferase activity, while this knocking down effect dropped significantly in the cells treated with transferrin solution. White bars represent luciferase activity (%) in comparison to untreated control cells (100%) obtained by using Luc-siRNA and gray bars represent activities obtained with siCONTROL. Data are mean and relative S.D. of 5 similar experiments. Asterisks indicate statistical significance comparing transferrin treated vs. untreated cells (*P<0.05; paired-sample t- test

To confirm the effect of Tf-PEI for increasing the transfection efficiency of OEI-HA10 through transferrin receptors on K562 cells, a competition assay using ferrous citrate in transferrin solution was performed (see material & methods). Again a very good knocking down (72%) was observed in the luciferase activity of K562 cells transfected with Luc-siRNA but not treated with ferrous citrate in transferrin solution, while the transfection efficiency dropped to 53% following use of ferrous citrate in transferrin solution 15 min prior to transfection (Figure 5).

## Discussion

SiRNA is considered to be a very promising tool for treatment of different diseases especially in cancer therapy, once efficient technologies for *in vivo* delivery are established (6). For the development of vectors applicable to *in vivo *siRNA transfer additional to intracellular barriers, physical restrictions and serological or immunological barriers have to be overcome. Ideally, transfection complexes would be highly soluble, specific for binding to the target cells, but inert against unspecific interactions with body fluids or tissues (12). Polyethylenimine (PEI) is a polycationic carrier for nucleic acids with intrinsic/inherent endosomal activity, mediating high DNA and siRNA transfection activity *in vitro *and also *in vivo*. *In vivo *application however is limited by significant toxicity and lack of biodegradability of the polymer (12-14). 

Using linear PEI in our study was completely ineffective for transfecting K562 cell line with siRNA. This result is similar to those reported by Philipp *et -al* and Meyer *et al* (13) for other (adherent) cell lines. 

In order to achieve target specificity we used cell-binding ligand, transferrin, coupled to PEI, combining the intrinsic activities of PEI with receptor-mediated uptake mechanisms. In previous studies, regarding DNA transfection, coupling of transferrin to PEI has been found to shift the optimal PEI N/P ratio to lower values, making this system more attractive for *in vivo *gene transfer. In addition the increased solubility of Tf-PEI–DNA complexes and their smaller particle size, which may facilitate the endocytotic event, might also be important factors (12, 15, 17,19).

In current study, applying Tf-PEI for transfecting K562 cells with siRNA was again ineffective and rather toxic.

To improve polycationic carriers for siRNA delivery that are as effective as PEI but less toxic, newly developed polymers were used based on cross-linking of low molecular weight OEI using various degradable linkages (16). In one study, OEI-HA10 (the structure containing ten hexyl acrylate residues per one OEI chain) was identified as a gene transfer carrier with comparable efficiency as PEI but lower toxicity. Importantly, unlike PEI, this polymer was found to be also effective for siRNA delivery *in vitro *(8).

Using OEI-HA10 in present study, up to 60% silencing was achieved, however, this effect was rather due to the high level of toxicity on K562 cells. This finding is in consistent with the study of Philipp *et al* in which, due to strong lytic activity, OEI-HA-10 formulations were relatively toxic in vitro. This will lead to the enhanced acute toxicity and lethality during *in vivo *applications (8). 

Co-formulation with lipophilic agents (DOPE liposome) was able to decrease the toxicity and increase transfection of the formulations due to integration of hydrophobic groups into the lipid bilayer. The optimal composition (OEI-HA-10/DOPE 1/2 w/w) induced the best knockdown in the study of Philipp *et al* (8). Applying such a co-formulation was also incapable of siRNA delivery into the K562 cells.

To test whether the relatively low efficiency of RNA transfer may be due to trapping and/or degradation of the RNA within the endosomal/lysosomal pathway, chloroquine, a drug known to alter lysosomal pH and thus to inhibit lysosomal degradation of macromolecules. It was therefore added to cells simultaneously with the OEI-HA10-siRNA complexes. It has been reported that 3-4 hours are sufficient for maximum DNA transfer activity (20). The aforementioned experiment was also not successful as it was highly toxic at any concentration studied. Correspondingly, Kircheis *et al* have been shown that chloroquine does not provide any further enhancement of the transferring-PEI system (12).

If the challenges through delivery of siRNA into K562 cells line are having low endosomal activity of OEI-HA10, utilizing PEI will be helpful due to its “proton-sponge effect”. This may promote the endosomal escape that was considered as a crucial step in the delivery process. Succinylated branched PEI showed far lower cytotoxicity compared to unmodified PEI and a high efficiency in siRNA-mediated knockdown in Neuro2ALuc cells *in vitro* (21). Hence, we utilized co-formulation of OEI-HA10 with sucPEI in order to transfect K562 cells with siRNA. This co-formulation was also ineffective and toxic.

Tietze *et al* in 2008 have shown that, optimiz- ed transferrin-conjugate/OEI/siRNA formulations displayed efficient knockdown efficiency in Neuro2A-EGFPLuc cells comparable with the non-shielded formulations (16).

Accordingly, we have applied OEI-HA10 (70%) plus Tf-PEI (30%) to further enhance the activity of endosomal escape plus receptor mediated endocytosis. This experiment was successful with strong suppression of luciferase activity and low toxicity in K562 cells after a short time (40 min). 

Transferrin competition assay was performed to evaluate the extent of transferrin effect on the knocking down effect. Figure 4 shows that free transferrin in the medium efficiently competed for the transferrin-PEI/OEI-mediated siRNA uptake, as observed by a 20% reduction of luciferase enzyme activity. Therefore, we have concluded that transfection complexes require binding to the transferrin receptor on the K562 cells for siRNA delivery. 

In one study it has been demonstrated that the presence of excess free Tf interferes with DNA uptake (17) and that up-regulation of the Tf receptor by agents like desferrioxamine (deferoxamine), increases the subsequent gene expression in K-562 cells (19). Virtually 100% of such cells take up and express a transferrinfected reporter gene (17). 

## Conclusion

In conclusion, in order to enhance the knocking down effect and reduce cytotoxicity on K562 cells, we recommend that K562 cell line transfection should be done using OEI-HA10/Tf-PEI/siRNA formulation, while the cells are pre-treated for 12 hr with 50 mM desferrioxamine, the procedure was used, to up-regulate transferrin receptors.
